# An Infrastructure for Integrated Electronic Health Record Services: The Role of XML (Extensible Markup Language)

**DOI:** 10.2196/jmir.3.1.e7

**Published:** 2001-03-17

**Authors:** Dimitrios G Katehakis, Stelios Sfakianakis, Manolis Tsiknakis, Stelios C Orphanoudakis

**Affiliations:** ^1^Center of Medical Informatics and Health Telematics Applications (CMI-HTA)Institute of Computer Science (ICS)Foundation for Research and TechnologyHeraklionCreteGreece; ^2^Department of Computer ScienceUniversity of CreteHeraklionCreteGreece

**Keywords:** Integrated Advanced Information Management Systems, Delivery of Healthcare, Medical Record Systems, Computerized, Hospital Information Systems, XML

## Abstract

**Background:**

The sharing of information resources is generally accepted as the key to substantial improvements in productivity and better quality of care. In addition, due to the greater mobility of the population, national and international healthcare networks are increasingly used to facilitate the sharing of healthcare-related information among the various actors of the field. In the context of HYGEIAnet, the regional health telematics network of Crete, an Integrated Electronic Health Record environment has been developed to provide integrated access to online clinical information, accessible throughout the island.

**Objectives:**

To make available comprehensive medical information about a patient by means of incorporating all the distributed and heterogeneous health record segments into an Integrated Electronic Health Record that can be viewed on-line through a unified user interface and visualization environment.

**Methods:**

The technological approach for implementing this Integrated Electronic Health Record environment is based on the HYGEIAnet Reference Architecture, which provides the necessary framework for the reuse of services, components, and interfaces. Seamless presentation of information is achieved by means of the Extensible Markup Language (XML), while its underlying capabilities allow for dynamic navigation according to personalized end-user preferences and authorities.

**Results:**

The Integrated Electronic Health Record environment developed in HYGEIAnet provides the basis for consistent and authenticated access to primary information over the Internet in order to support decision-making. Primary information is always kept at the place where it has been produced, and is maintained by the most appropriate clinical information system, contrasting traditional store and forward techniques, or centralized clinical data repositories.

**Conclusions:**

Since documents are much more easily accessible rather than data inside a database, Extensible Markup Language has the potential of becoming a very cheap technology provided, of course, that the underlying Healthcare Information Infrastructure exists. XML can be introduced incrementally and its implementation is completely transparent to the end user.

## Introduction

Parts of patients' medical records are located in all the places where they have received clinical services (eg, community doctors, primary care, and secondary care). All of these segments, which are related to personal healthcare delivery and well being, reside in places that are disparate and, in most cases, not directly accessible. Moreover, a number of restrictive policies do not allow personal, sensitive clinical information to be carried outside the corresponding organization's boundaries, while the healthcare providers continue to maintain detailed and confidential notes about their cases. This is true even when healthcare providers use electronic clinical record systems and communication between them is by electronic means. Although the World Wide Web (WWW) provides the means for global access to all kinds of information, personal health information still remains fragmented and not directly accessible in a unified way.

Any Integrated Electronic Healthcare Record (I-EHR) environment should be capable of handling these issues and provide uniform ways for accessing authentic, physician-generated, patient record information that is physically located in different clinical information systems. Furthermore, it needs to provide fast and authorized on-line access to longitudinal views of each patient's personal health record, in order to allow for the timely delivery of health care. Such an environment is expected to allow patients to become more actively involved in the monitoring and assessment of their own wellness. At this point, the main reason driving the need for integrated access to clinical information is information sharing. Issues that need to be resolved on the way towards providing integrated solutions are mainly focused around patient identification, interoperability among cooperating software components and the involved clinical information systems, and all the security related medico-legal issues.

## Methods

The technological approach for implementing the I-EHR environment is based on the HYGEIAnet Reference Architecture (HRA), which provides the necessary framework for the reuse of services, components, and interfaces [1]. At the middleware level, these services include: authorization, naming, messaging, terminology, semantic mapping, and other metadata services, as well as services for the management of medical acts, patient identification, and clinical data location [2].

**Figure 1:**
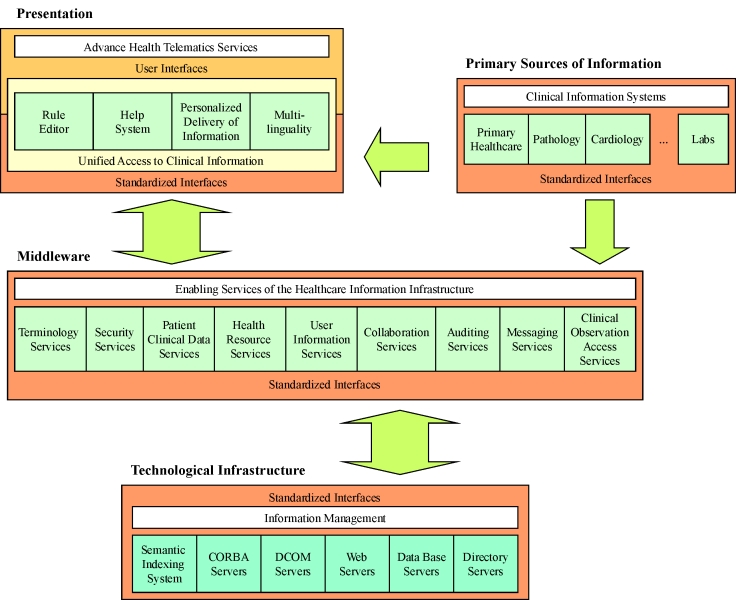
A two-dimension view of the multi-level architectural framework for the I-EHR environment of HYGEIAnet

The HRA applications and services model that was used provides a logical paradigm of the relationships between applications, end-user services and the underlying middleware enabling services. At the bottom layer, generic services and tools (eg, data bases and directories), the Internet and software component infrastructures (eg, Common Object Request Broker Architecture [CORBA], Distributed Common Object Model [DCOM], or Common Object Model Plus [COM+]) form the technological infrastructure for storing and managing information. Autonomous clinical information systems are the information sources to be integrated. These information sources can be accessed by means of a number of alternative interfaces (eg, Web/ Open Data Base Connectivity [ODBC] and CORBA). On top, the presentation layer provides the end-user with the means for accessing advanced I-EHR services and supporting activities in the various areas of the organization. Visualization can be delivered by means of, for example, the Web or Wireless Access Protocol (WAP) over a number of different possible devices (Personal Computers [PCs], handheld computers, mobile phones, etc.). The heart of the whole I-EHR environment, and core of the underlying Healthcare Information Infrastructure (HII), consists of middleware services that provide the mechanisms for information provision, filtering, and fusion. Figure 1 depicts how the required components for building the I-EHR are structured.

The currently available execution architecture is based on CORBA interfaces (for data acquisition, patient identification, semantic mapping and messaging), X.500/ Light Directory Access Protocol ( LDAP) (for security services, user profiles, patient clinical information, and healthcare resources), dedicated Structured Query Language (SQL)/ODBC-LDAP gateways (for accessing primary information and maintaining up-to-date indexing), and the Extensible Markup Language (XML) (to sustain the collected clinical information in a consistent way). Primary information is usually kept on commercial data base management systems, and this is expected to continue in the years to come. A key strength of existing databases is their ability to allow complex queries about clinical information that is kept in single data repositories. On the other hand, the emerging directory technology promises enhanced integrity offering personalized user environments, simplified service and application configurations, security service integration, and improved bandwidth allocation. Key strengths of the emerging directory technology are its distribution provisions and fast lookup based on name. The International Telecommunication Union's (ITU) X.500 and LDAP are the most promising approaches for building global directories [3]. Well-documented interfaces. expressed in the Interface Definition Language (IDL) associated with the integration framework of CORBAmed [4], provide basic support for interoperability among computer systems. This is essential, particularly in large hospitals, where many different kinds of computers have been installed and cannot be changed. The results of the CORBAmed efforts in standardizing IDLs has been to influence the design of interfaces developed worldwide due to its strong industrial support. Adopted models and architectures can be easily used by any alternative implementation (eg, DCOM, or COM+) or combinations.

Recently, the Extensible Markup Language (XML) has gained great attention and is becoming the preferred language for data interchange over the WWW. Its origins are in the Standard Generalized Markup Language (SGML), but in comparison, XML is simpler [5]. It looks like the HyperText Markup Language (HTML), but it's stricter and more generic since anyone can define the vocabulary intended for use. It is well defined, and there is emerging technology and tools for authoring, validating and presenting. XML offers freedom in using user-defined vocabularies, while the content is forced to conform to strict grammars (Document Type Descriptions [DTDs], XML schemas) that define how the tags can be mixed. The only thing that needs be described inside an XML document is the content, together with the component parts of the document, and not its presentation. Since its raw format is plain text, any XML document can easily be exchanged over well-known protocols such as the HyperText Transfer Protocol (HTTP) or the File Transfer Protocol (FTP), making it a very flexible platform for structuring and exchanging information.

## Results

The I-EHR is a front-end to an EHR indexing service, managed by the Patient Clinical Data Directory (PCDD) [2] which indices both structured and unstructured information that is provided by cooperating information systems, without imposing any constraint on their internal operation or their interface beyond the medical encounter level. At its current implementation, the main objective of the I-EHR environment is to deliver an encounter-centered view of the patient's EHR. It utilizes the available CORBA interfaces to provide a consistent way to locate, access and transmit secure information about a patient's EHR segments. Throughout any regional setting, these segments are maintained by a wide diversity of existing, autonomous, networked clinical information sources having different internal structures (database schemata) and different vocabularies to describe the notions they use.

References to recorded data are obtained and used to retrieve actual information by means of the Object Management Group's (OMG) Clinical Observation Access Service (COAS) implementations. COAS seems to be generic and simple, yet powerful, expressing the clinical observations and the relationship between them, composition being the most common. On the other hand, the terms used to describe and identify these observations may come from different coding schemes, and so a terminology service implementation is also necessary. This terminology service is responsible for concept mapping and translation between coding schemes. OMG's Terminology (or Lexicon) Query Service (TQS) is used at this point to provide both conceptual mappings among the different clinical information systems available and the coding schemes they use for recording clinical findings. This is a requirement in order for the existing information to be capable of providing comparable patient data among different institutions.

**Figure 2:**
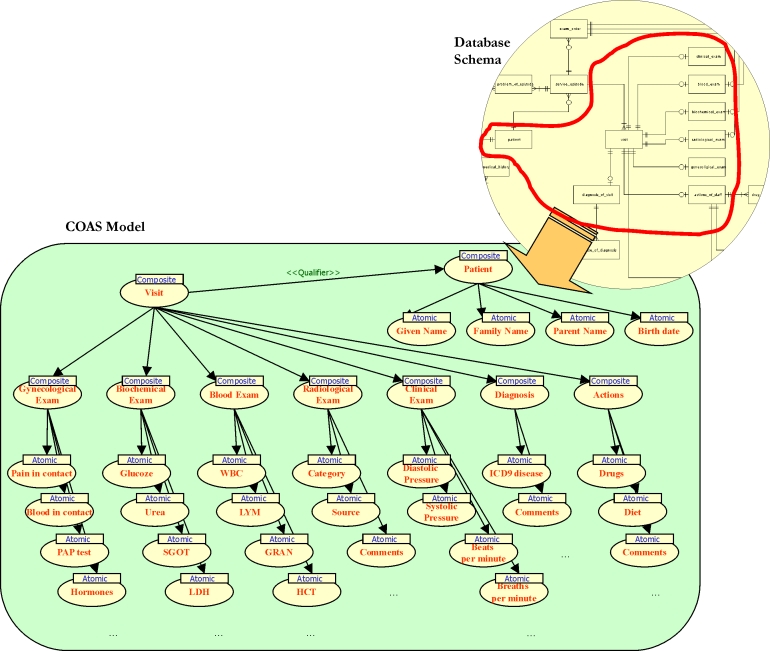
Example of database schema mapping to the COAS observation structure

In this context, a generic mapping between observations and attributes of database tables has been deployed. Composite observations have been mapped to database views while atomic observations have been mapped to attributes. The composite observations contain other observations, and this composition in the database is implemented through links and references from one table to another (Figure 2). Observations that are contained or related to another observation are, in turn, views that reference other tables and so forth. This recursion ends when an atomic observation is found and, if this is the case, the value of an attribute is retrieved. There is a specific mapping for each type of clinical information source, and each mapping follows a coding scheme accessible through a terminology server so that a client can "understand" the semantics of the information returned to it. The actual COAS implementation is the same for all information systems as long as they store their information in a relational database system. When moving from an information source to another, the actual implementation remains the same and the only thing that changes is the mapping from internal database relations to observations, provided that these information sources store their data in relational databases.

As far as the Graphical User Interface (GUI) for accessing the I-EHR environment is concerned, apart from the lifeline view of all the available encounters of the patient, a number of alternative views are currently supported: a per-clinical system view of the encounter's history, as well as the traditional tabular view of old generation GUIs. When requested, primary information is collected and presented to the end user by initiating remote COAS servers. The COAS data returned need further transformation in order to be properly presented to the user. The underlying data model supported by the Patient Clinical Data Directory (PCDD) is based on the Subjective Objective Assessment Plan (SOAP) model that originates from the primary healthcare domain [6].

In this context, XML has not only been used to describe the COAS observation data in a human readable format but also to be the central point of the transformation process. The composition and recursion concepts that are an integral part of the COAS representation of clinical observations are inherently supported in XML. An XML tag represents each COAS observation. If this is a composite observation, then this tag contains other tags that represent the component observations, and so on, until an atomic observation is reached. An atomic observation is a different tag that can have a "value" attribute or "parsed character data" as its content.

**Figure 3:**
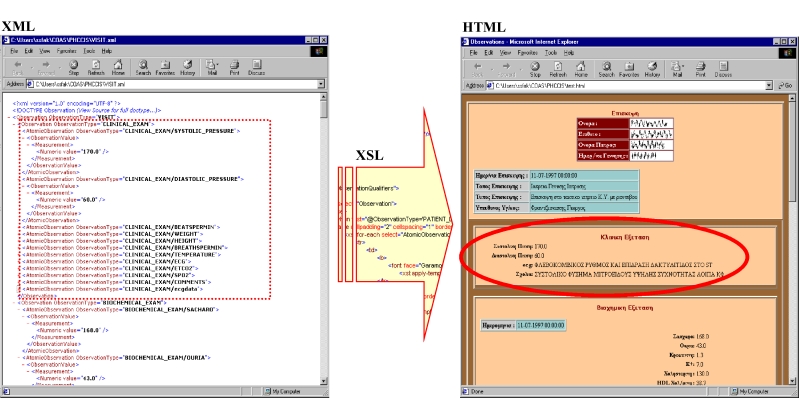
XML to HTML transformation by means of XSL

The COAS results are represented in XML through a DTD that has been developed (available in Appendix 1). Having been represented in XML, the clinical information can be transformed to many other formats, such as HTML and the Portable Data Format (PDF), using the Extensible Stylesheet Language (XSL). Such an example is depicted in Figure 3.

In a potential use scenario, the end user needs to locate and access clinical information about a specific patient. The system forwards this request to all known or existing COAS servers and collects their responses. These clinical observations are transformed to XML and then transformed again to a number of HTML pages. These pages can be presented to the user sorted chronologically, and the user can browse going back and forth in time. Alternatively, XML clinical data can be grouped by type and then transformed to HTML pages where the user can view a patient's clinical information categorized according to personal preferences (eg, all biochemical exams together).

Currently a COAS server and a COAS client have been implemented using the Java Programming Language [7] with CORBA as the communication infrastructure. Java is a Web application accessible through the WWW. The client side, which is responsible for collecting the COAS results of a user's query, saves them in XML format and transforms the XML data to HTML. The implementation has been developed using Java Servlet technology [8] and the Jakarta Tomcat 3.2 servlet container [9]. The system is stable enough for practical use. However, the deployment of such a system also requires other fully implemented components, such as the OMG's Person Identification Service (PIDS) to ensure unique identification of patients, TQS to manage different nomenclatures, and the Resource Access Decision (RAD) service to impose security policies. These components are required not only as far as COAS is concerned, but also in the general context of HYGEIAnet and are work-in-progress, partly implemented today.

## Discussion

HYGEIAnet builds on a regional healthcare information infrastructure to improve the quality and accessibility of health care and to enable the delivery of integrated health care services. It provides the information and services that are the foundation for accountability, continuous improvement to health care, and better understanding of the determinants of the population's health. The design of HYGEIAnet is based on the existing regional healthcare system in Greece. Its goal is to serve local the local population, regardless of whether they are patients, healthcare professionals, researchers, or managers. The I-EHR environment, as it has been developed and set up, provides a decentralized view of the patient's medical record, by dynamically composing information that resides in a variety of heterogeneous clinical information systems. Under a secure Internet/ Intranet environment, the full personal health history can be rapidly collected and composed totally transparently and sent to the authorized health professional (the Internet/ Intranet is not limited in capacity). In addition, maintaining electronic health record information is extremely economical to the very end users and consumers of the system (the citizens themselves), since the cost is transferred to the healthcare practitioners keeping primary information and to telecom operators and ISPs maintaining regional or national networks. The I-EHR, as used in the current context, is "virtual" in that it provides a uniform view of data (metadata) possibly configured to work differently at different locations.

Users seek selective information following specific paths, depending on their personal preferences, so it is expected that the I-EHR concept will eventually lead to a uniform applications and services environment. Since electronic records can provide much easier navigational facilities, navigational issues are expected to become even more important in the future, mainly because end-users require interfaces which are similar in look and feel.

The lack of a standardized interface for accessing clinical objects has forced the current implementation to follow an open architecture approach that utilizes the best available technologies for accessing clinical multimedia data. It is indeed a fact that information systems use different technologies and terms for accessing the same clinical objects. CORBAmed currently leads the definition of the interoperable specification effort that can support activities related to directly accessing a greater variety of healthcare information. XML provides the appropriate technology and makes up the most convenient vehicle towards a common format for delivering and presenting information content. Elaboration of the standard DTD logical structure and related XML infrastructure will make information personalization flexible and generic enough to adapt to various types of users and client devices. Since documents (accompanied of course by the physician's signature) are much more easily accessible than data inside a database, XML has the potential of becoming a very cheap technology, provided of course, that the underlying HII exists. XML can be introduced incrementally, and its implementation is completely transparent to the end user. One of the main advantages of this approach is the support of context searching capabilities.

Currently, major organizations like Health Level Seven (HL7) [10], the Comité Européen de Normalisation Technical Committee 251 [11], and the American Society for Testing and Materials [12] work in modeling the electronic health record and are expected to provide useful DTDs for the healthcare domain. In the case of HL7 Clinical Document Architecture (previously known as 'Patient Record Architecture'), defined as "a document markup standard for the structure and semantics of exchanged clinical documents," documents are encoded in XML and can be put in a hierarchy of increasing strictness and detail. At the HL7 level, the only HL7 DTD currently available is one DTD, which uses only blocks of free text and coded entries to represent the patient's record. Unfortunately, very few results about the standardization of DTDs currently exist worldwide for the medical domain; no need mentioning best practice examples, and significant effort ought to be paid towards that direction.

## Appendix 1

<!ELEMENT Query (Observation)*>
<!ATTLIST Query TimeofQuery CDATA #REQUIRED
                WhoAsk CDATA #REQUIRED
                SelectedQuery CDATA #REQUIRED
                GeographicRegion CDATA #REQUIRED
                TimeRange CDATA #REQUIRED
                Gender CDATA #REQUIRED
                Age CDATA #REQUIRED
<!ELEMENT Observation (AtomicObservation | CompositeObservation)>
<!ATTLIST Observation Patient_ID CDATA #REQUIRED
                Information_System CDATA #REQUIRED
                Visit_ID CDATA #REQUIRED>
<!ELEMENT CompositeObservation ((AtomicObservation | CompositeObservation)*
                       ,ObservationReference *, ObservationQualifier *)>
<!ATTLIST CompositeObservation   ObservationType CDATA #REQUIRED
                                 ObservationTime CDATA #IMPLIED>
<!ELEMENT AtomicObservation (ObservationValue,ObservationReference*, ObservationQualifier *)>
<!ATTLIST AtomicObservation    ObservationType CDATA #REQUIRED
                               ObservationTime CDATA #IMPLIED>
<!ELEMENT ObservationValue ((PlainText | NoInformation | CodeElement | LooselyCodeElement | Curve
                     MultiMedia | DateTime | Measurement | TechnologyInstanceLocator),
                     ObservationQualifier *)>
<!ELEMENT Plaintext EMPTY>
<!ATTLIST PlainText  Value CDATA #REQUIRED
                     language CDATA #IMPLIED>
<!ELEMENT NoInformation EMPTY>
<!ATTLIST NoInformation reason CDATA #REQUIRED>
<!ELEMENT CodeElement EMPTY>
<!ATTLIST CodeElement value CDATA #REQUIRED
                      printName CDATA #IMPLIED>
<!ELEMENT LooselyCodeElement EMPTY>
<!ATTLIST LooselyCodeElement text CDATA #REQUIRED
                     codingSchemeID CDATA #REQUIRED
                     versionID CDATA #REQUIRED>

<!ELEMENT Curve EMPTY>
<!ATTLIST Curve values CDATA #REQUIRED
                xUnits CDATA #IMPLIED
                yUnits CDATA #IMPLIED
<!ELEMENT Multimedia EMPTY>
<!ATTLIST Multimedia header CDATA #REQUIRED>
<!ELEMENT DateTime EMPTY>
<!ATTLIST DateTime value CDATA #REQUIRED
                   relationalOperator CDATA #IMPLIED
                   accuracy CDATA #IMPLIED
                   accuracycontext CDATA #IMPLIED
                   accuracyUnit CDATA #IMPLIED>
<!ELEMENT Measurement EMPTY>
<!ATTLIST Measurement NumericValue CDATA #REQUIRED
                      units CDATA #IMPLIED>
<!ELEMENT TechnologyInstanceLocator EMPTY>
<!ATTLIST TechnologyInstanceLocator protocol CDATA #REQUIRED
                                    address CDATA #REQUIRED>
<!ELEMENT ObservationQualifier (QualifiedBy)*>
<!ATTLIST ObservationQualifier ObservationQualifierType CDATA #REQUIRED>
<!ELEMENT QualifiedBy (ObservationQualifier)+>
<!ELEMENT ObservationReference EMPTY>
<!ATTLIST ObservationReference ObservationReferenceType CDATA #REQUIRED
                    ObservationReferenceName CDATA #REQUIRED
                    Patient_Id CDATA #REQUIRED
                    Information_System CDATA #REQUIRED
                    Visit_Id CDATA #REQUIRED
                    ObservationTime CDATA #IMPLIED>

## Abbreviations

CMI-HTA: Centerfor Medical Informatics and Health TelematicsApplications
COAS: ClinicalObservation Access Service
CORBA: CommonObject Request Broker Architecture
COM+: CommonObject Model Plus
DCOM: Distributed Common Object Model
DTD: Document Type Description
EHR: Electronic Health Record
FORTH: Foundation for Research and Technology - Hellas
FTP: FileTransfer Protocol
GUI: Graphical User Interface
HII: Healthcare Information Infrastructure
HRA: HYGEIAnet Reference Architecture
HTML: HyperText Markup Language
HTTP: HyperText Transfer Protocol
I-HER: Integrated Electronic Health Record
IDL: Interface Definition Language
ITU: International Telecommunication Union
LDAP: LightDirectory Access Protocol
ODBC: OpenData Base Connectivity
OMG: ObjectManagement Group
PC: PersonalComputer
PCDD: PatientClinical Data Directory
PDF: PortableData Format
PIDS: PersonIdentification Service
RAD: ResourceAccess Decision
SGML: StandardGeneralized Markup Language
SQL: Structured Query Language
SOAP: Subjective Objective Assessment Plan
TQS: Terminology Query Service
WAP: WirelessAccess Protocol
WWW: WorldWide Web
XML: Extensible Markup Language
XSL: Extensible Stylesheet Language

## References

[1] Tsiknakis M, Chronaki CE, Kapidakis S, Nikolaou C, Orphanoudakis SC (1997). An Integrated Architecture for the Provision of Health Telematic Services based on Digital Library Technologies. Int J Dig Libr.

[2] Katehakis DG, Lelis P, Karabela E, Tsiknakis M, Orphanoudakis SC (2000). An Environment for the Creation of an Integrated Electronic Health Record in HYGEIAnet, the Regional Health Telematics Network of Crete. Proc TEPR.

[3] International Telecommunication Union.

[4] The Object Management Group.

[5] The World Wide Web Consortium.

[6] Potamias G, Tsiknakis M, Katehakis D, Karabela E, Moustakis V, Orphanoudakis S (2000). Role-based access to patients clinical data: the InterCare approach in the region of Crete. Stud Health Technol Inform.

[7] The Java Programming Language.

[8] The Java Servlet Technology.

[9] The Jakarta Tomcat servlet container.

[10] Health Level Seven, SGML/XML Special Working Group Patient Record Architecture (PRA).

[11] Comité Européen de Normalisation (CEN), Technical Committee 251, Working Group I Information Models.

[12] American Society for Testing and Materials (ASTM), subcommittee E31.25.

